# Supplementary material for: Par complex cluster formation mediated by phase separation
Source: Nat Commun. 2020 May 8;11:2266. doi: 10.1038/s41467-020-16135-6 (PMC7211019; doi:10.1038/s41467-020-16135-6)
Supplement: Supplementary file 1 — Supplementary Information [file 41467_2020_16135_MOESM1_ESM.pdf]

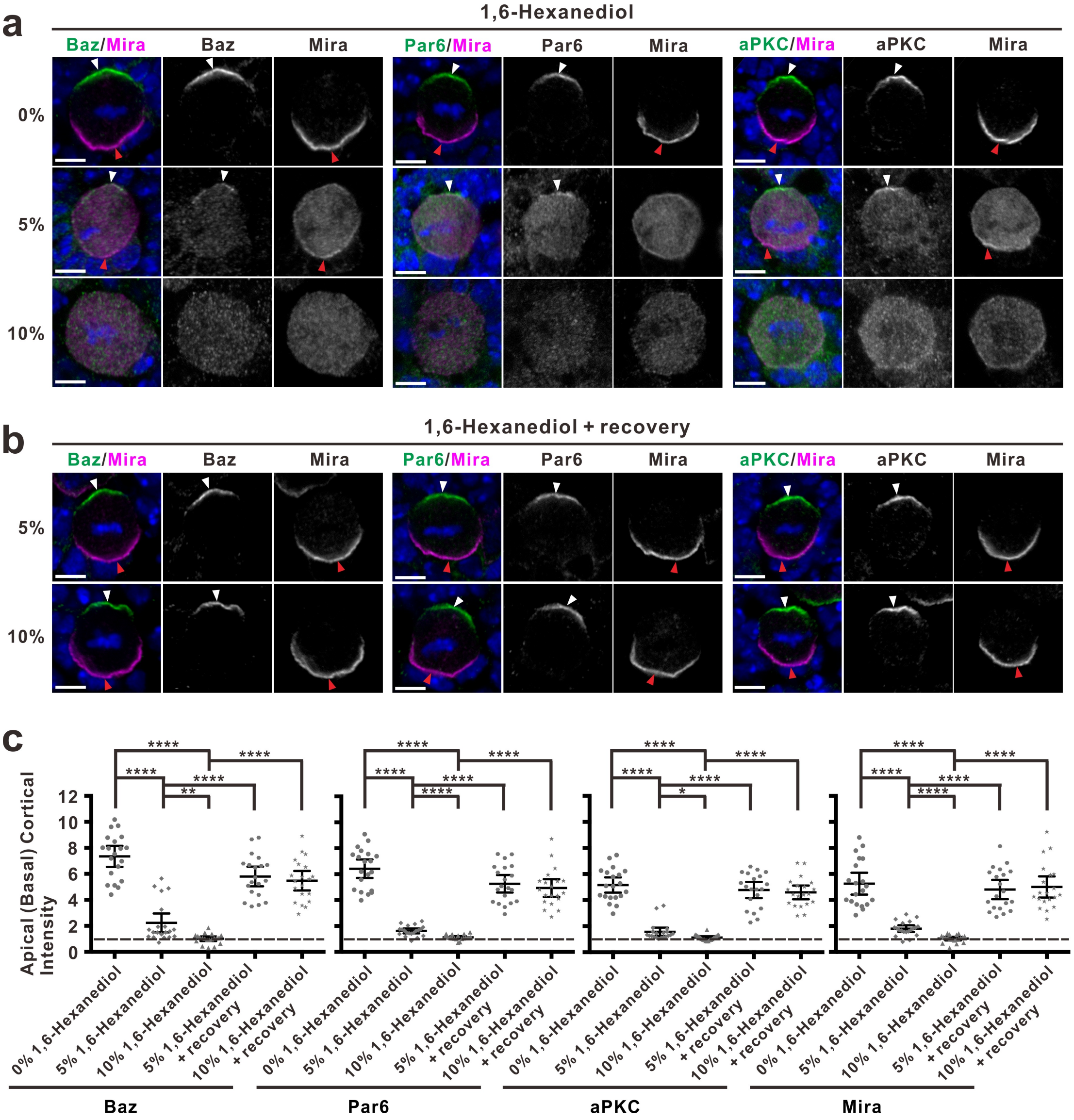

Supplementary Figure 1. Phase separation of the Par complex was reversible *in vivo*. a, Representative images of metaphase larval NBs (showing various ACD proteins including Baz, Par6, aPKC and Mira) from control, 5% or 10% 1,6-Hexanediol treated brains, respectively. **b**, Representative images of metaphase larval NBs showing various ACD proteins after removal of 1,6-Hexanediol. White arrowheads point to the apical cortex whereas red ones indicate the basal cortex. Topro-3 in blue in (**a**) and (**b**). Scale bars, 5um. **c**, Quantification of the ACI (Apical or Basal Cortical Intensity) for various ACD proteins. Experiments were performed three times independently with similar results. n=20 neuroblasts collected from 15 larval brains for each genotype over 3 independent experiments. For all the statistical data, mean ± 95% confidence interval (CI) is shown. ns, not significant, $*p < 0.05$, $**p < 0.01$, $***p < 0.001$ and $****p < 0.0001$ using one-way analysis of variance (ANOVA) with Tukey's multiple comparison test. Source data are provided as a Source Data file.

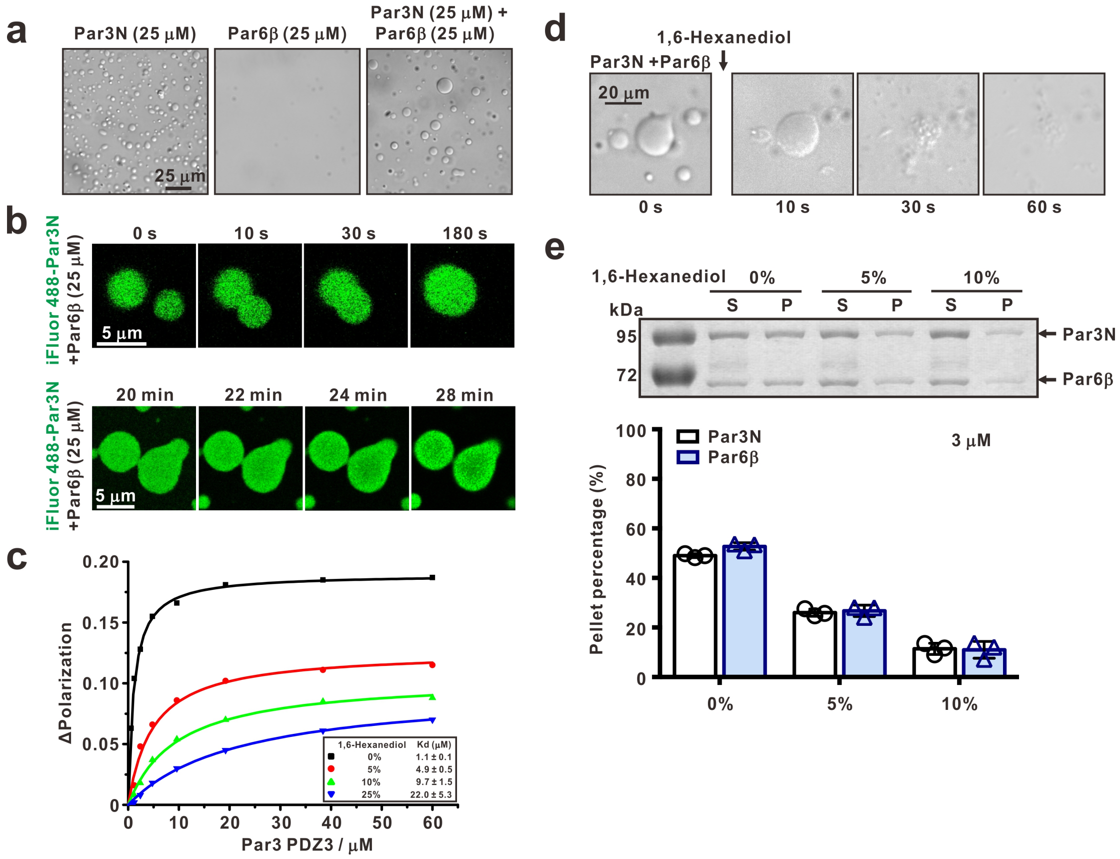

Supplementary Figure 2. Phase separation of the Par3N/Par6β Complex. a, Isolated Par6β solution is clear under light microscope at room temperature whereas isolated Par3N solution can form numerous small droplets. Mixing the two proteins promoted the droplets fusing into much larger ones. The images were acquired 3.5 min and onward after mixing. **b**, Fusion assay of the droplets formed by mixing iFluor488-Par3N with Par6β. **c**, Fluorescence polarization-based measurements of the binding affinities between Par3 PDZ3 and Par6β PBM peptide with various concentrations of 1,6-hexanediol. **d**, Pre-formed Par3N/Par6β (25 µM) droplets were rapidly dispersed after adding 10% 1,6-Hexanediol. The arrow refers to the time point of adding 1,6-Hexanediol to the mixture. **e**, Pre-formed Par3N/Par6β (3 µM) droplets could be reversed to aqueous phase by 1,6-Hexanediol in a dose dependent manner. The final concentration of 1,6-Hexanediol is indicated. Experiments were performed three times independently with similar results. Data are expressed as mean ± SD. Source data are provided as a Source Data file.

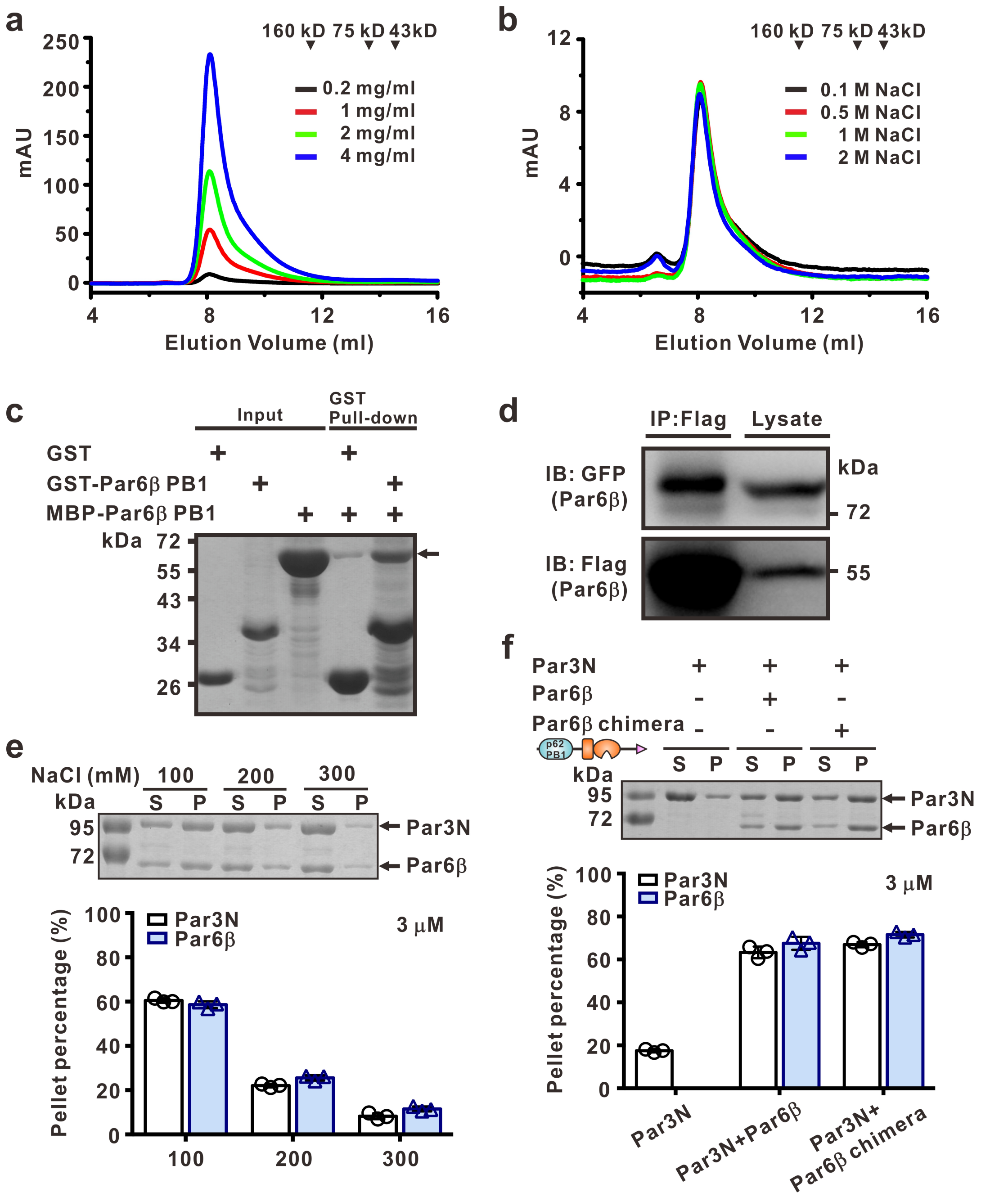

Supplementary Figure 3. Par6β PB1 forms oligomer in vitro. a&b, Analytical gel filtration profiles of the Par6 PB1 at different protein concentrations (**a**) or under different salt concentrations (**b**). **c**, GST pull-down assay showing that GST-tagged Par6β PB1 bound to MBP-tagged Par6β PB1. **d**, Co-IP assay showing that Flag-tagged full-length Par6β could co-precipitate with GFP-Par6β. **e**, LLPS of Par3N and Par6β complex is regulated by salt concentration. **f**, The promotion effect of Par6β on Par3N LLPS is dependent on its PB1 oligomerization, which could be retained when PB1 was replaced with another self-associating domain p62 PB1 (Par6β chimera). Experiments were performed three times independently with similar results. Data are expressed as mean ± SD. Source data are provided as a Source Data file.

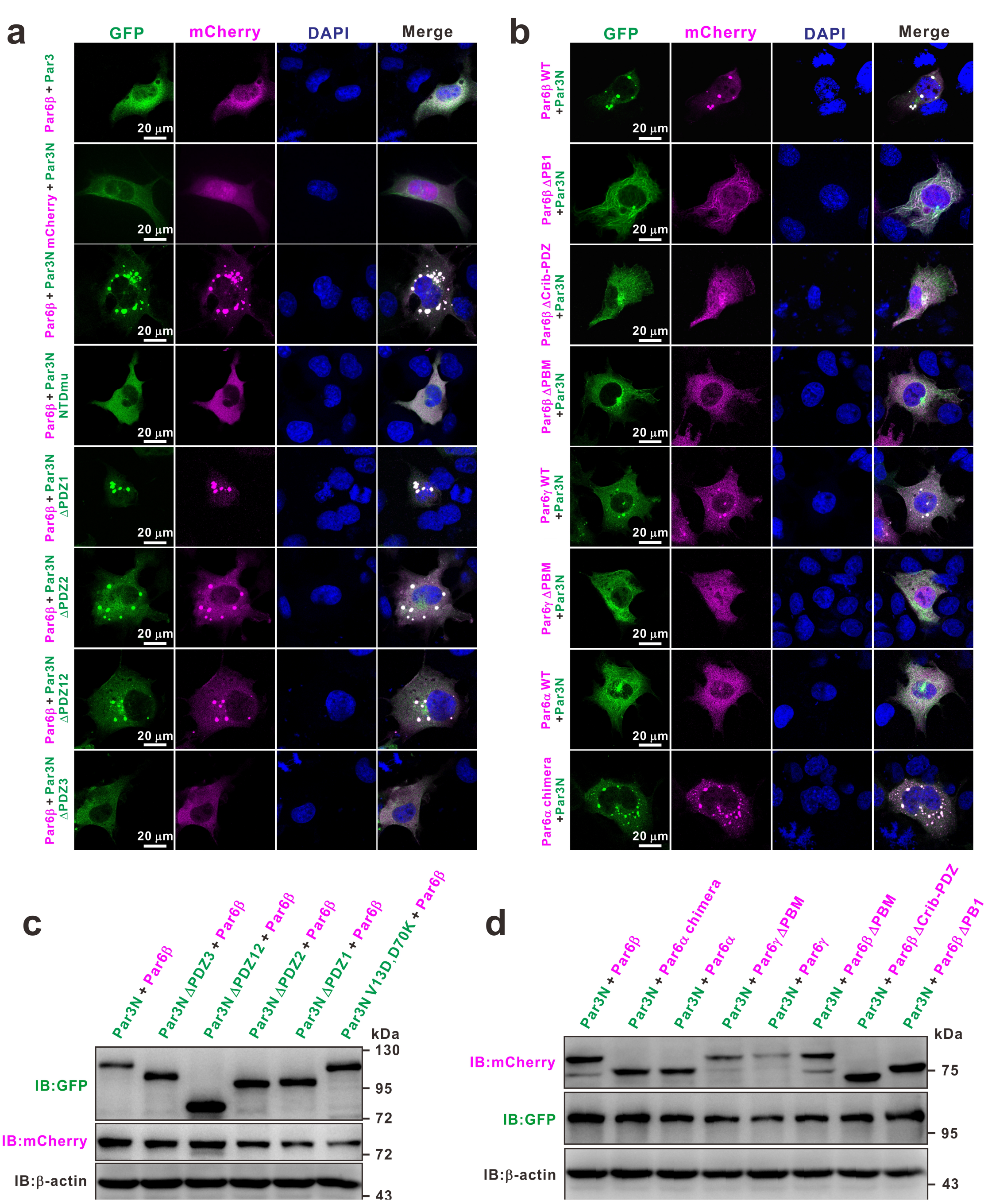

Supplementary Figure 4. The Par3N/Par6β complex forms condensed liquid phase in living cells. **a**, Representative images showing expression of GFP-Par3 WT or various mutants with mCherry-Par6β or mCherry vector (Mock) in COS7 cells, and some cells exhibit multiple bright puncta containing both fluorophores. **b**, Representative images showing expression of GFP-Par3N with various mCherry-Par6β constructs. **c**, Expression of Par3N WT and various mutants with Par6β in COS7 cells in (**a**) were detected by Western-blotting assay. **d**, Expression of various Par6 fragments with Par3N in COS7 cells in (**b**) were detected by Western-blotting assay. Experiments were performed three times independently with similar results. Source data are provided as a Source Data file.

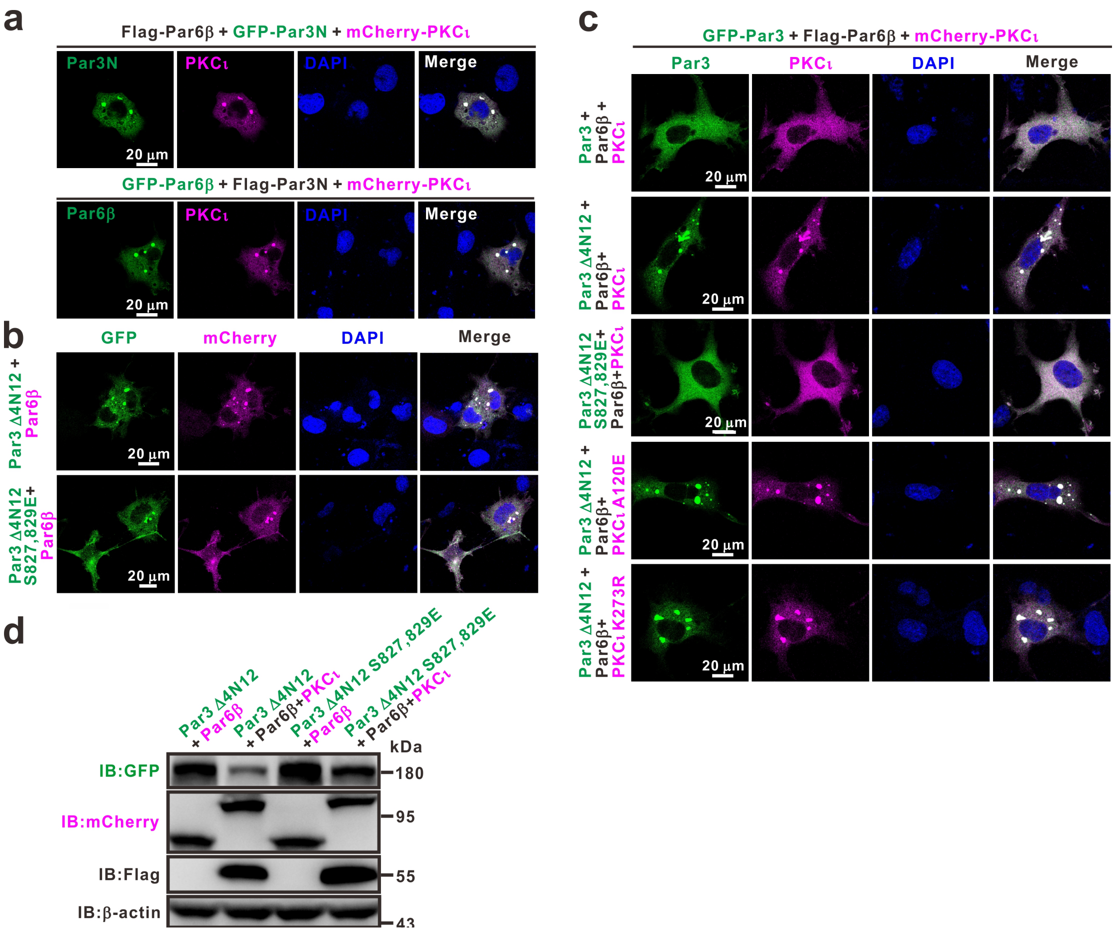

Supplementary Figure 5. The activity of aPKC is suppressed in the Par complex. **a**, Representative images showing co-localization and condensation of Par3N, Par6β, and PKCι in COS7 cells. **b**, Representative images showing co-expression of GFP-Par3 Δ4N12 WT or the aPKC phospho-mimetic S827,829E mutant with mCherry-Par6β in COS7 cells. **c**, Representative images showing co-expression of GFP-Par3 WT, Δ4N12 or the phospho-mimetic Δ4N12 S827,829E with Flag-Par6β, and mCherry-PKCι WT, the constitutively active A120E, or the kinase dead K273R mutant in COS7 cells. **d**, Expression of Par3 Δ4N12 or Par3 Δ4N12 S827,829E with flag/mCherry-tagged Par6β and mCherry-tagged PKCι in COS7 cells in (**b**) and (**c**) were detected by Western-blotting assay. Experiments were performed three times independently with similar results. Source data are provided as a Source Data file.

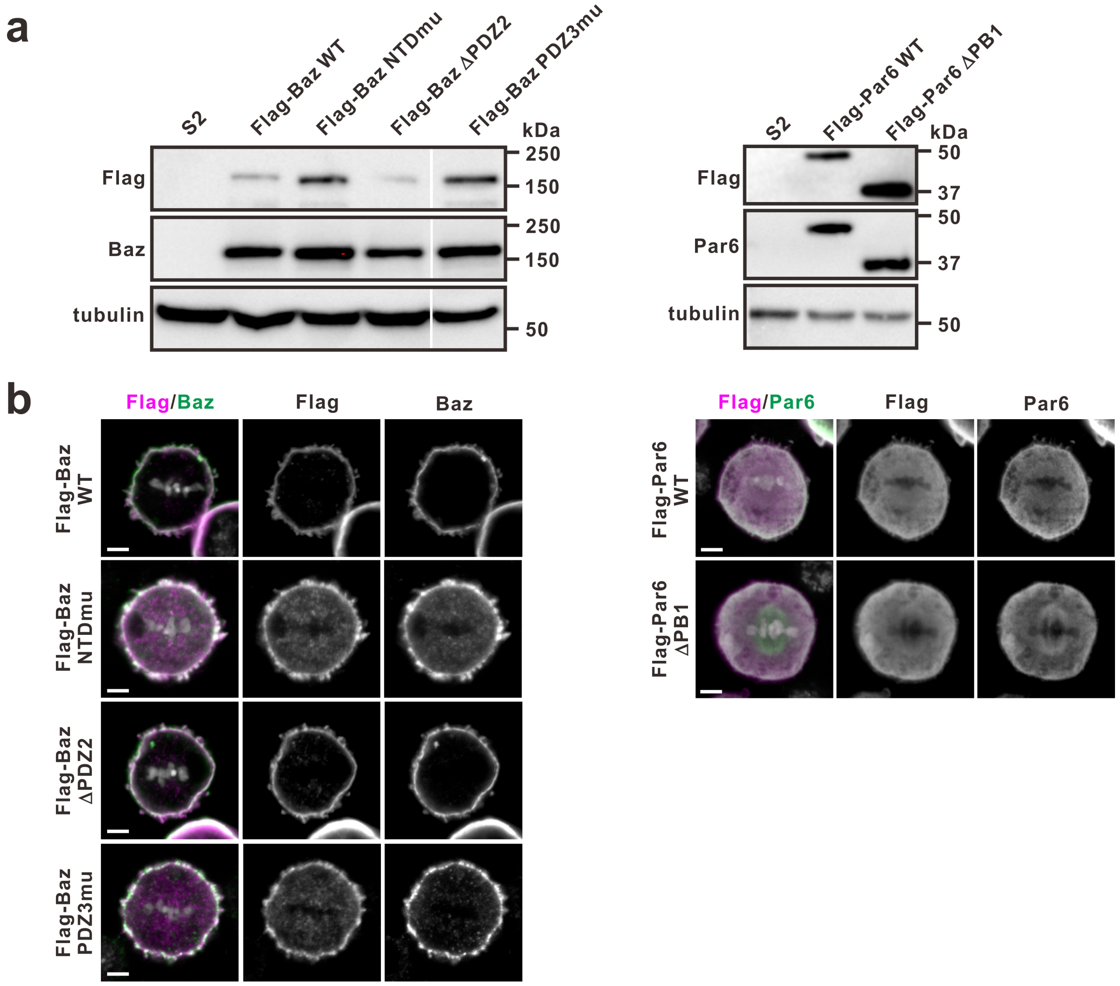

Supplementary Figure 6. Expression of Flag-tagged Baz and Par6 variants in *Drosophila* S2 cells. Western-blotting (**a**) or staining (**b**) showing representative images of S2 cells expressing various Flag-tagged Baz or Par6 fragments. Scale bars, 5 μm for all the S2 cell images. Experiments were performed three times independently with similar results. Source data are provided as a Source Data file.

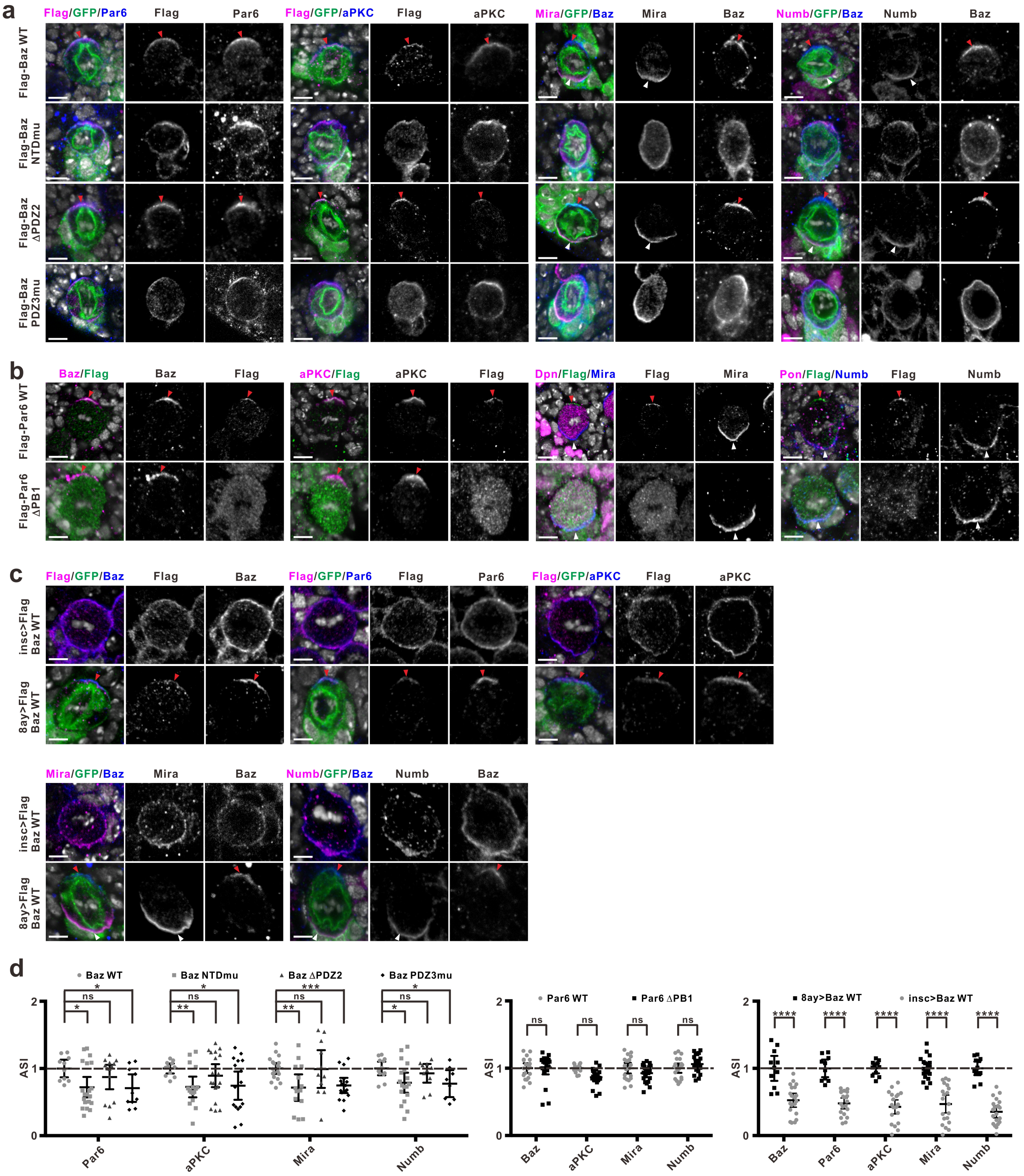

Supplementary Figure 7. Baz/Par6 phase separation is required for polarized localization of apical and basal proteins during ACD of *Drosophila* type I larval NBs. a&b, Representative images showing larval NB expressing *Flag-Baz WT* or *mutant* variants (**a**, by an *actin* “Flip-out” system marked by GFP, n= 20 neuroblasts collected from 60 larval brains for each genotype over 3 independent experiments) or *Flag-Par6 WT* or Δ*PB1* (**b**, by a *UAS/gal4* system driven by *insc-gal4*, n= 20 neuroblasts collected from 15 larval brains for each genotype over 3 independent experiments) in wild type NBs. Endogenous Baz/Par6 (as well as the Flag-tagged proteins), and aPKC are apically localized, whereas Mira, Pon and Numb are basally localized in wild-type NBs expressing *Flag-Baz WT*, *Flag-Baz ΔPDZ2* or *Flag-Par6 WT*. However, in wild-type NBs expressing *Flag-Baz NTDmu* or *PDZ3mu*, Flag-Baz proteins, and endogenous apical (Baz, Par6 and aPKC) and basal (Mira and Numb) proteins diffuse on the whole cortex (with some cytoplasmic localization). Interestingly, in wild-type NBs expressing *Flag-Par6 ΔPB1*, though endogenous Baz and aPKC seem correctly apical localized, Mira and Numb form slightly broader crescent on the basal

cortex compared with the wild-type NBs expressing *Flag-Par6 WT*. **c**, Ectopic expression of exogenous *Flag-Baz* (driven by *insc-gal4*, n=20 neuroblasts collected from 15 larval brains for each genotype over 3 independent experiments) in wild type larval NBs leads to defective localizations of both apical proteins including Baz, Par6, aPKC and basal fate determinants Mira and Numb, whereas wild type larval NBs expressing exogenous *Flag-Baz* (by an *actin* "Flip-out" system marked by GFP, n=11 neuroblasts collected from 30 larval brains for each genotype over 3 independent experiments) exhibit normal asymmetric localization of these proteins. **d**, Statistical quantification for (**a**-**c**). For all the statistical data, mean ± 95% CI is shown. ns, not significant, $*p < 0.05$, $**p < 0.01$, $***p < 0.001$ and $****p < 0.0001$ using one-way analysis of variance (ANOVA) with Tukey's multiple comparison test. ToPro-3 is in white. White arrowheads point to basal cortex whereas red arrowheads indicate apical cortex. Scale bars, 5 µm. Source data are provided as a Source Data file.

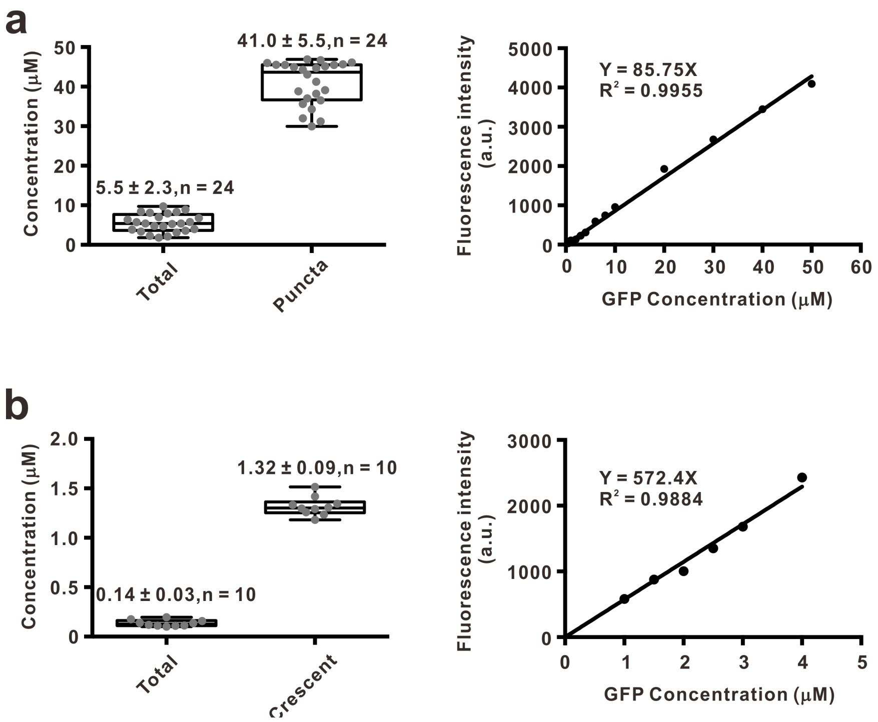

Supplementary Figure 8. Quantification of protein concentrations in cells. a, Concentrations of Par3N overexpressed in COS7 cells. The right panel is calibration curve showing the relationship between GFP concentration and GFP fluorescence intensity. The black curve shows the linear regression analysis of the fluorescence intensities versus protein concentrations. **b**, Concentrations of endogenous Baz in NBs. The right panel is calibration curve showing the relationship between GFP concentration and GFP fluorescence intensity. The black curve shows the linear regression analysis of the fluorescence intensities versus protein concentrations. In this figure, data are presented as box-and-whisker plots. Median values (middle bars); 25th and 75th percentile (boxes); whiskers indicate 1.5x the interquartile ranges; grey circles indicate individual data points. Source data are provided as a Source Data file.

Supplementary Table 1. Constructs used in this study

Par3 (Baz)	
Par3 FL (1-1337)	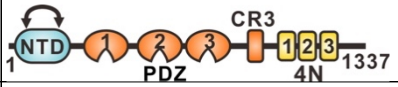
Par3 Δ4N12 (1-932+1221-1337)	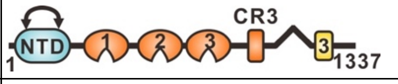
Par3N (1-685)	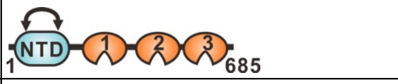
Par3 NTD (1-82)	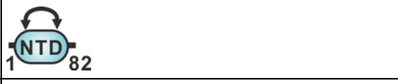
Par3 PDZ1 (263-381)	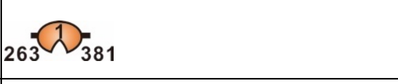
Par3 PDZ2 (382-581)	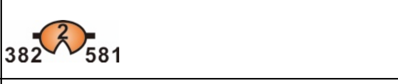
Par3 PDZ3 (582-685)	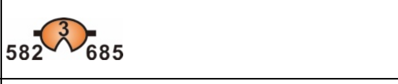
Par3N NTDmu (1-685 V13D D70K)	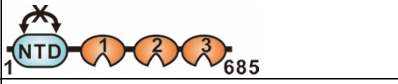
Par3N ΔNTD (83-685)	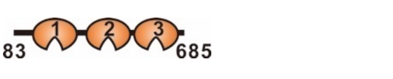
Par3N ΔPDZ1 (1-262+382-685)	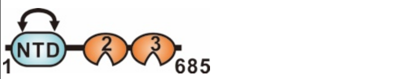
Par3N ΔPDZ2 (1-381+582-685)	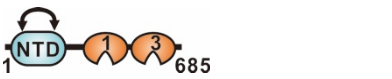
Par3N ΔPDZ3 (1-582)	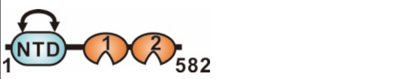
Par3N ΔPDZ12 (1-262+582-685)	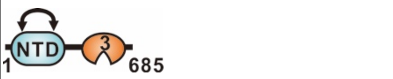
FUS_L-Par3N (1-214+83-685)	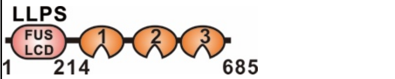
FUS_S-Par3N (1-141+83-685)	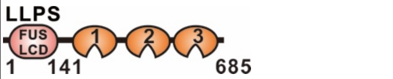
Baz FL (1-1464)	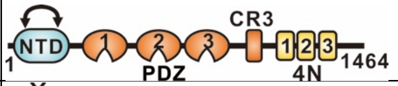
Baz NTDmu (1-1464 L13D D68K)	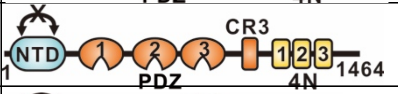
Baz ΔPDZ2 (1-378+515-1464)	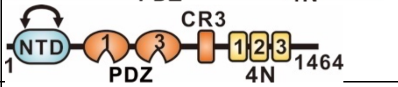
Baz PDZ3mu (1-1464 G634, 636A)	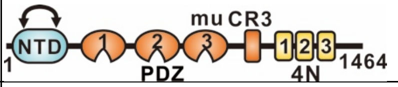
Baz ΔNTD (1-42+91-1464)	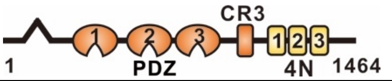

FUS_L-Baz (1-42+1-214+91-1464)	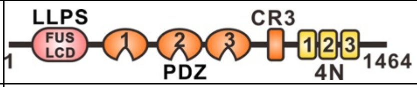
FUS_S-Baz (1-42+1-141+91-1464)	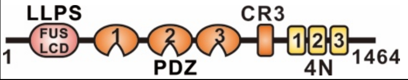
Par6 (Par6)	
Par6α FL (1-346)	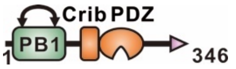
Par6α chimera (1-342+368-371)	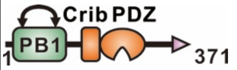
Par6β FL (1-371)	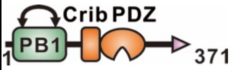
Par6β PB1 (1-100)	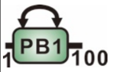
Par6β Crib-PDZ (100-253)	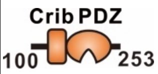
Par6β PBM (253-371)	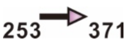
Par6β PB1-Crib-PDZ (1-253)	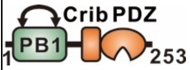
Par6β Crib-PDZ-PBM (100-371)	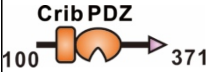
Par6β ΔPB1 (100-371)	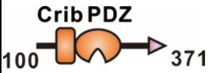
Par6β ΔCrib PDZ (1-100+253-371)	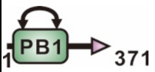
Par6β ΔPBM (1-367)	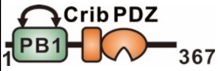
Par6β chimera (1-102+100-371)	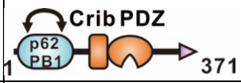
Par6γ FL (1-382)	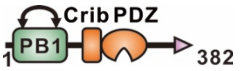
Par6γ ΔPBM (1-378)	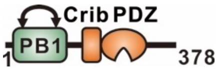
Par6 FL (1-351)	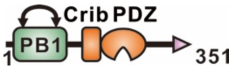
Par6 ΔPB1 (102-351)	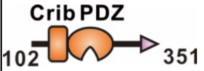
PKCι (aPKC)	
PKCι FL (1-586)	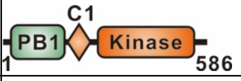
PKCι PB1 (16-99)	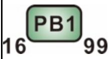

aPKC FL (1-606)	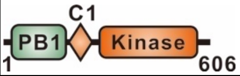

Supplementary Table 2. Primers used in this study

For generating constructs expressing Par3, Par6 and aPKC	
Par3 FL-F	CGGATATCCATGAAAGTGACCGTG
Par3 FL-R	TTGCGGCCGCTTAGGAGTAGAAGGGCCG
Par3N-F	CGGAATTCATGAAAGTGACCGTG
Par3N-R	TTGCGGCCGCTTAGCTGATCCGCCTCGC
Par3 NTD-F	CGGAATTCATGAAAGTGACCGTG
Par3 NTD-R	CCCTCGAGTTACTGCTCATCAAACAC
Par3 PDZ1-F	CGGAATTCTCCTCTCTGAGTGCC
Par3 PDZ1-R	TTGCGGCCGCTTAGCGGCCAGGGGAATA
Par3 PDZ2-F	CGGGATCCGACAGCCACTGTGTG
Par3 PDZ2-R	TTGCGGCCGCTTAGTCAGGTGTGAGAAC
Par3 PDZ3-F	CGGGATCCGGTACCAGGGAGTTT
Par3 PDZ3-R	CCAAGCTTTTAGTCAGGTGTGAGAAC
Par3N NTDmu-F1	CGGAATTCATGAGCGATAAAATT
Par3N NTDmu-R1	GC GTCGACTTAGCTGATCCGCCTCGC
Par3N NTDmu-F2	GGGAGGACCCGGGTGGACGTGCCGTGCGGAGAT
Par3N NTDmu-R2	ATCTCCGCACGGCACGTCCACCCGGGTCCTCCC
Par3N NTDmu-F3	CTCTGTGACGTTGCCAAAGACAAAGACAGACTG
Par3N NTDmu-R3	CAGTCTGTCTTTGTCTTTGGCAACGTCACAGAG
Par3N ΔNTD-F	CGGAATTCGATCCCCACCATGGA
Par3N ΔNTD-R	GCGTCGATTAGCTGATCCGCCTCGC
Par3N ΔPDZ1-F1	CGGAATTCATGAGCGATAAAATT
Par3N ΔPDZ1-R1	GCTGTCGGGGCTGAAATTCTCCAGTCCAGT
Par3N ΔPDZ1-F2	ACTGGACTGGAGAATTTCAGCCCCGACAGC
Par3N ΔPDZ1-R2	GCGTCGACTTAGCTGATCCGCCTCGC
Par3N ΔPDZ2-F1	CGGAATTCATGAGCGATAAAATT
Par3N ΔPDZ2-R1	AAACTCCCTGGTACCGCGGCCAGGGGAATA
Par3N ΔPDZ2-F2	TATTCCCCTGGCCGCGGTACCAGGGAGTTT
Par3N ΔPDZ2-R2	GCGTCGACTTAGCTGATCCGCCTCGC
Par3N ΔPDZ3-F	CGGAATTCATGAGCGATAAAATT
Par3N ΔPDZ3-R	TTGCGGCCGCTTAACCGTCAGGTGTGAG
Par3N ΔPDZ12-F1	CGGAATTCATGAGCGATAAAATT
Par3N ΔPDZ12-R1	AAACTCCCTGGTACCATTCTCCAGTCCAGT
Par3N ΔPDZ12-F2	ACTGGACTGGAGAATGGTACCAGGGAGTTT
Par3N ΔPDZ12-R2	GCGTCGACTTAGCTGATCCGCCTCGC
FUS_L-Par3N-F1	CCGAATTCGAGCTCCGTCGACAAATGGCCTCAAACGATTATACCC
FUS_L-Par3N-R1	ATGGTGGGGATCTCCACGGTCCTGCTGTCCA
FUS_L-Par3N-F2	ACCGTGGAGATCCCCACCATGGAGGAGA
FUS_L-Par3N-R2	GTGGATCGCTCGAGTGCGGCCGCTTAGCTGATCCGCCTCGCC

FUS$_S$-Par3N-F1	CCGAATTCGAGCTCCGTCGACAAATGGCCTCAAACGATTATACCC
FUS$_S$-Par3N-R1	TCTCCTCCATGGTGGGGATCTTGCTGCTGT
FUS$_S$-Par3N-F2	ACAGCAGCAAGATCCCCACCATGGAGGAGA
FUS$_S$-Par3N-R2	GTGGATCGCTCGAGTGCGGCCGCTTAGCTGATCCGCCTCGCC
Par3 Δ4N12-F1	CGGATATCCATGAAAGTGACCGTG
Par3 Δ4N12-R1	TTGTCTTGGCAGGGAGGACTTGTCAATGGCGGCTC
Par3 Δ4N12-F2	TCCCTGCCAAGACAAAGCAGGAAGAATGCCAGCTC
Par3 Δ4N12-R2	TTGCGGCCGCTTAGGAGTAGAAGGGCCG
Par3 Δ4N12 S827,829E-F1	CGGATATCCATGAAAGTGACCGTG
Par3 Δ4N12 S827,829E-R1	TTCTTCCATTTCCTGGCGTCCAAATCCTTC
Par3 Δ4N12 S827,829E-F2	CAGGAAATGGAAGAAAAACGCACAAAGCAA
Par3 Δ4N12 S827,829E-R2	TTGCGGCCGCTTAGGAGTAGAAGGGCCG
Par6α FL-F	CGGAATTCATGGCCAGGCCGCAG
Par6α FL-R	CCAAGCTTTTAAGCGGATTCAGCCTC
Par6α chimera-F	CCAAGCTTCGATGGCCAGGCCGCAG
Par6α chimera-R	CGGGATCCTTAACCGTCTTCCTCTAAGCCATTCCCCCAGCC
Par6β FL-F	CGGGATCCATGAACCGCGGCCAC
Par6β FL-R	CCAAGCTTTTATAATGTGATGATGGT
Par6β PB1-F	CGGGATCCATGAACCGCGGCCAC
Par6β PB1-R	CCCTCGAGTTAGTCAGCTTCTTCCTT
Par6β Crib-PDZ-F	CGGAATTCGACTACAGTGCCTTT
Par6β Crib-PDZ-R	TTGCGGCCGCGTTCCTCTGGTTGGC
Par6β PBM-F	CGGAATTCAACAACGTGGTGCGC
Par6β PBM-R	CCAAGCTTTTATAATGTGATGATGGT
Par6β PB1-Crib-PDZ-F	CGGAATTCATGAACCGCGGCCAC
Par6β PB1-Crib-PDZ-R	TTGCGGCCGCGTTCCTCTGGTTGGC
Par6β Crib-PDZ-PBM-F	CGGAATTCATGAACCGCGGCCAC
Par6β Crib-PDZ-PBM-R	TTGCGGCCGCTTATAATGTGATGATGGTACCGTC
Par6β ΔPB1-F	CGGAATTCGACTACAGTGCCTTT
Par6β ΔPB1-R	TTGCGGCCGCTTATAATGTGATGATGGTACCGTC
Par6β ΔCrib PDZ-F1	CGGAATTCATGAACCGCGGCCAC
Par6β ΔCrib PDZ-R1	GCGCACCACGTTGTTGTCAGCTTCTTCCTT
Par6β ΔCrib PDZ-F2	AAGGAAGAAGCTGACAACAACGTGGTGCGC
Par6β ΔCrib PDZ-R2	TTGCGGCCGCTTATAATGTGATGATGGTACCGTC
Par6β ΔPBM-F	CGGGATCCATGAACCGCGGCCAC
Par6β ΔPBM-R	CCAAGCTTTTAGGTACCGTCTTCCTC
Par6β chimera-F1	CGGAATTCATGAACCGCGGCCAC
Par6β chimera-R1	AAAGGCACTGTAGTCACTGTAGTCAGCTTC
Par6β chimera-F2	GAAGCTGACTACAGTGACTACAGTGCCTTT
Par6β chimera-R2	TTGCGGCCGCTTATAATGTGATGATGGTACCGTC

Par6γ FL-F	CGGGATCCATGAACCGGAGTTTT
Par6γ FL-R	CCAAGCTTTTAGAGTGTAATGGCTGG
Par6γ ΔPBM-F	CCAAGCTTCGATGAACCGGAGTTTT
Par6γ ΔPBM-R	CGGGATCCTTATGGCCCATGTTCCTC
PKCι FL-F	CCAAGCTTCGATGTCCCACACGGTC
PKCι FL-R	CGGGATCCTTAGACACACTCTTCTGC
PKCι PB1-F	CGGAATTCCAGGTCCGGGTGAAA
PKCι PB1-R	CCCTCGAGTTAACATGGAAATACATG
For generating transgenic flies expressing Baz and Par6 variants	
Baz-flag-Not1-F	CACCGCGGCCGCATGGATTACAAGGATGATGATGATAAGA AGGTCACCGTCTGCTTCGGCG
Baz-Kpn1-R	CACAGGTACCTCACACCTTGGAGGCGTGTGGC
Par6-flag-Not1-F	CACCGCGGCCGCATGGATTACAAGGATGATGATGATAAGT CGAAGAACAAGATAAACACAAC
Par6-Xba1-R	CACCTCTAGACTACAAATGCAGCACTCCATC
Par6-ΔPB1-flag-EcoR1-F	CACCGAATTCATGGATTACAAGGATGATGATGATAAGAAG ACAAAGGCGCCATCGATATC
Par6-ΔPB1-Xho1-R	CACCCTCGAGCTACAAATGCAGCACTCCATC
Baz NTDmu-F1	CCGAATTCATGAAGGTCACCGTCTGCTTCG
Baz NTDmu-R1	TTGTCGACTTACACCTTGGAGGCGTGTGGC
Baz NTDmu-F2	GGCGACGTTCGCATTGATGTTCCCTGTGGTTCC
Baz NTDmu-R2	GGAACCACAGGGAACATCAATGCGAACGTCGCC
Baz NTDmu-F3	GTCCGCGACGTGGCCAAAGATCGGGAGCAGATA
Baz NTDmu-R3	TATCTGCTCCCGATCTTTGGCCACGTCGCGGAC
Baz ΔPDZ2-F1	CCGAATTCATGAAGGTCACCGTCTGCTTCG
Baz ΔPDZ2-R1	CGCTGGCATGCCCCTTTGCTCCTGAACCTG
Baz ΔPDZ2-F2	CAGGTTCAGGAGCAAAGGGGCATGCCAGCG
Baz ΔPDZ2-R2	TTGTCGACTTACACCTTGGAGGCGTGTGGC
Baz PDZ3mu-F1	CCGAATTCATGAAGGTCACCGTCTGCTTCG
Baz PDZ3mu-R1	TCCCTTCACACTGACAGCCAGTGCGGCCTTTTCGGTGTC
Baz PDZ3mu-F2	GACACCGAAAAGGCCGCACTGGCTGTCAGTGTGAAGGG A
Baz PDZ3mu-R2	TTGTCGACTTACACCTTGGAGGCGTGTGGC
Baz ΔNTD-F1	CCGAATTCATGAAGGTCACCGTC
Baz ΔNTD-R1	CGATGCTCCATCTCCATCGGGCTTGCCGGC
Baz ΔNTD-F2	GCCGGCAAGCCCGATGGAGATGGAGCATCG
Baz ΔNTD-R2	TTGTCGACTTACACCTTGGAGGCGTGTGGC
For generating CRISPR CAS9 mediated GFP-Baz ΔNTD knocked-in fly	
gRNA-F:	TATATAGGAAAGATATCCGGGTGAACTTCAATCCAGGAGT TCCGCAAGGGTTTTAGAGCTAGAAATAGCAAG
gRNA-R	ATTTTAACTTGCTATTTCTAGCTCTAAAACCGATCCGGATG

	ATTGTGTCCGACGTTAAATTGAAAATAGGTC
BazL-F	TCGCTGAAGCAGGTGGAATTCGCGAAAAATAGCCGAAATTATTG
BazL-R	CTCACCATATCGGGCTGTAAGTTTAAAAAATACA
GFP-F	TTACAGCCCGATATGGTGAGCAAGGGCGAGG
GFP-R	TCCATCTCCCTTGTACAGCTCGTCCATGCC
BazR-F	AGCTGTACAAGGGAGATGGAGCATCGGGC
BazR-R	GGTGTGCATATGTCCGCGGCCGCTCGATGGCCGCACCTCGT
For generating CRISPR CAS9 mediated GFP-FUS_L-Baz and GFP-FUS_S-Baz knocked-in fly	
gRNA -F	same as for GFP-Baz ΔNTD
gRNA -R	same as for GFP-Baz ΔNTD
BazFUSL-F	TCGCTGAAGCAGGTGGAATTCGACATCGAAGAAACCATGTGAAGA
BazFUSL-R	CTCACCATATCGGGCTGTAAGTTTAAAAAATACA
GFPlinker-F	TTACAGCCCGATATGGTGAGCAAGGGCGAGG
GFPlinker-R	TTGAGGCCATACCTGATCCAGCTGCTGCTGAACCTGGAGCTCCCTTGTACAGCTCGTCCATGCC
FUS-F	TGGATCAGGTATGGCCTCAAACGATTATACCC
FUS_S-R	CCTTGCTGCTGTCCACCATAGCT
FUS_L-R	ATGCTCCATCTCCGCCTCCACGGTCCTGCTGTCCA
FUS_SBazR-F	TATGGTGGACAGCAGCAAGGCGGAGATGGAGCATCG
FUS_LBazR-F	TGGAGGCGGAGATGGAGCATCG
FUSBazR-R	GGTGTGCATATGTCCGCGGCCGCGCGGGTGGGTGACACCGT
