# Supplementary material for: Par complex cluster formation mediated by phase separation
Source: Nat Commun. 2020 May 8;11:2266. doi: 10.1038/s41467-020-16135-6 (PMC7211019; doi:10.1038/s41467-020-16135-6)
Supplement: Supplementary file 3 — Description of Additional Supplementary Files [file 41467_2020_16135_MOESM3_ESM.pdf]

Description of Additional Supplementary Files

File Name: Supplementary Movie 1
Description: Time-dependent fusion of small GFP-Par3N/mCherry-Par6β puncta into larger ones in living COS7 cells, related to Fig. 2c.

File Name: Supplementary Movie 2
Description: Fluorescence signal recovery of GFP-Par3N in a COS7 cell co-expressing with Flag-Par6β, related to Fig. 2d.

File Name: Supplementary Movie 3
Description: Fluorescence signal recovery of GFP-Par6β in a COS7 cell co-expressing with HA-Par3N, related to Fig. 2e.

File Name: Supplementary Movie 4
Description: Time-dependent fusion of small iFluorTM 488-Par3N droplets into larger ones in vitro, related to Fig. 3e.

File Name: Supplementary Movie 5
Description: Time-dependent fusion of small iFluorTM 488-Par3N/Cy3-Par6β droplets into larger ones in vitro, related to Fig. 3f.

File Name: Supplementary Movie 6
Description: Fluorescence signal recovery of iFluorTM 488-Par3N in vitro, related to Fig. 3g.

File Name: Supplementary Movie 7
Description: Fluorescence signal recovery of iFluorTM 488-Par3N in the Par6β-bound droplets in vitro, related to Fig. 3g.

File Name: Supplementary Movie 8
Description: 1,6-Hexanediol-induced dispersion of the Par3N/Par6β droplets, related to Supplementary Fig. 2d.
