# Supplementary material for: Par complex cluster formation mediated by phase separation
Source: Nat Commun. 2020 May 8;11:2266. doi: 10.1038/s41467-020-16135-6 (PMC7211019; doi:10.1038/s41467-020-16135-6)
Supplement: Supplementary file 12 — Reporting Summary [file 41467_2020_16135_MOESM12_ESM.pdf]

nature research

Corresponding author(s): Wenyu Wen

Last updated by author(s): Apr 1, 2020

Reporting Summary

Nature Research wishes to improve the reproducibility of the work that we publish. This form provides structure for consistency and transparency in reporting. For further information on Nature Research policies, see Authors & Referees and the Editorial Policy Checklist .

Statistics

For all statistical analyses, confirm that the following items are present in the figure legend, table legend, main text, or Methods section.

n/a	Confirmed	
☐	☒	The exact sample size (*n*) for each experimental group/condition, given as a discrete number and unit of measurement
☐	☒	A statement on whether measurements were taken from distinct samples or whether the same sample was measured repeatedly
☐	☒	The statistical test(s) used AND whether they are one- or two-sided<br>*Only common tests should be described solely by name; describe more complex techniques in the Methods section.*
☒	☐	A description of all covariates tested
☒	☐	A description of any assumptions or corrections, such as tests of normality and adjustment for multiple comparisons
☐	☒	A full description of the statistical parameters including central tendency (e.g. means) or other basic estimates (e.g. regression coefficient) AND variation (e.g. standard deviation) or associated estimates of uncertainty (e.g. confidence intervals)
☐	☒	For null hypothesis testing, the test statistic (e.g. *F*, *t*, *r*) with confidence intervals, effect sizes, degrees of freedom and *P* value noted<br>*Give P values as exact values whenever suitable.*
☒	☐	For Bayesian analysis, information on the choice of priors and Markov chain Monte Carlo settings
☒	☐	For hierarchical and complex designs, identification of the appropriate level for tests and full reporting of outcomes
☒	☐	Estimates of effect sizes (e.g. Cohen's *d*, Pearson's *r*), indicating how they were calculated

Our web collection on statistics for biologists contains articles on many of the points above.

Software and code

Policy information about availability of computer code

Data collection	No software was used for data collection.
Data analysis	Data analysis was carried out in ImageJ 1.51s, PHASER 1.13-2998, COOT 0.8.9.1,HKL2000 0.98.703x, PHENIX 1.13-2998, Adobe photoshop CC 2018, Prism 6

For manuscripts utilizing custom algorithms or software that are central to the research but not yet described in published literature, software must be made available to editors/reviewers. We strongly encourage code deposition in a community repository (e.g. GitHub). See the Nature Research guidelines for submitting code & software for further information.

Data

Policy information about availability of data

All manuscripts must include a data availability statement. This statement should provide the following information, where applicable:

- Accession codes, unique identifiers, or web links for publicly available datasets
- A list of figures that have associated raw data
- A description of any restrictions on data availability

All data supporting the findings of this study are available within the article and its Supplementary Information files or from the corresponding author upon request. Coordinates of the crystal structure of Par3 PDZ3/Par6β PBM complex have been deposited in the Protein Data Bank (http://www.rcsb.org/) under the accession code 6JUE. FlyBase dataset is available online at http://flybase.org.

Field-specific reporting

Please select the one below that is the best fit for your research. If you are not sure, read the appropriate sections before making your selection.

Life sciences study design

All studies must disclose on these points even when the disclosure is negative.

Sample size	For NB sample size, we follow previous publications in this field with a minimum of 10 NB counted (see reference listed below). For baz and par6 mutants which are known to be difficult for clone generation, we used a minimal number of 5 as published data.<br>1 Samuels, T. J., Jarvelin, A. I., Ish-Horowicz, D. & Davis, I. Imp/IGF2BP levels modulate individual neural stem cell growth and division through myc mRNA stability. eLife 9, doi:10.7554/eLife.51529 (2020).<br>2 Sullivan, L. F., Warren, T. L. & Doe, C. Q. Temporal identity establishes columnar neuron morphology, connectivity, and function in a Drosophila navigation circuit. eLife 8, doi:10.7554/eLife.43482 (2019).<br>3 Pahl, M. C., Doyle, S. E. & Siegrist, S. E. E93 Integrates Neuroblast Intrinsic State with Developmental Time to Terminate MB Neurogenesis via Autophagy. Current biology : CB 29, 750-762.e753, doi:10.1016/j.cub.2019.01.039 (2019).<br>4 Froldi, F. et al. Histidine is selectively required for the growth of Myc-dependent dedifferentiation tumours in the Drosophila CNS. The EMBO journal 38, doi:10.15252/embj.201899895 (2019).<br>5 Metivier, M. et al. Dual control of Kinesin-1 recruitment to microtubules by Ensconsin in Drosophila neuroblasts and oocytes. Development (Cambridge, England) 146, doi:10.1242/dev.171579 (2019).
Data exclusions	No data was excluded.
Replication	All assays were repeated three times showing similar results
Randomization	Our samples were collected randomly.We adapted a double-blind method to assign the samples to the experimental group.
Blinding	We were blinded to group allocation during data collection and analysis.

Reporting for specific materials, systems and methods

We require information from authors about some types of materials, experimental systems and methods used in many studies. Here, indicate whether each material, system or method listed is relevant to your study. If you are not sure if a list item applies to your research, read the appropriate section before selecting a response.

Materials & experimental systems

n/a	Involved in the study	
☐	☒	Antibodies
☐	☒	Eukaryotic cell lines
☒	☐	Palaeontology
☒	☐	Animals and other organisms
☒	☐	Human research participants
☒	☐	Clinical data

Methods

n/a	Involved in the study	
☒	☐	ChIP-seq
☒	☐	Flow cytometry
☒	☐	MRI-based neuroimaging

Antibodies

Antibodies used	anti-Flag (ABclonal, AE005), goat anti-mouse antibody (ABclonal, AS003), goat anti-rabbit antibody (ABclonal, AS014), Mouse anti-Flag antibody (Sigma, F1804), rabbit anti-Mira (generated in our lab); guinea-pig anti-Dpn (generated in our lab); rabbit anti-Baz (generated in our lab); rabbit anti-Par6 (generated in our lab); rabbit anti-aPKC C20 (Santa Cruz Biotechnologies, SG-216-G), chicken anti-GFP (Abcam, ab13970); rabbit anti-Pon (generated in our lab); rabbit anti-Numb (a gift from Xiaohang Yang)
Validation	anti-Flag (ABclonal, AE005), goat anti-mouse antibody (ABclonal, AS003), and goat anti-rabbit antibody (ABclonal, AS014) have been validated in the following reference:<br>Wang, Z., Liu, Z., Chen, X., Li, J., Yao, W., Huang, S., Gu, A., Lei, Q.-Y., Mao, Y., and Wen, W. “A multi-lock inhibitory mechanism for fine-tuning enzyme activities of the HECT family E3 ligases” Nat. Commun. (2019) 10, 3162.<br><br>Mouse anti-Flag antibody (Sigma, F1804) and have been validated in the following reference:<br>Shan, Z., Tu, Y., Yang, Y., Liu, Z., Zeng, M., Xu, H., Long, J., Zhang, M., Cai, Y., and Wen, W. “Basal condensation of Numb and Pon complex via phase transition during Drosophila neuroblast asymmetric division” Nat. Commun. (2018) 9, 737.<br><br>Rabbit anti-Mira antibody has been validated in the following reference:<br>Chai P, Liu Z, Chia W, Cai Y. (2013) Hedgehog signaling acts with the temporal cascade to promote neuroblast cell cycle exit. PLoS Biology 11: e1001494<br><br>Guinea-pig anti-Dpn antibody has been validated in the following reference:<br>Jia M, Shan Z, Yang Y, Liu C, Li J, Luo ZG, Zhang M, Cai Y, Wen W and Wang W. (2015) The structural basis of Miranda-mediated Staufen localization during Drosophila neuroblast asymmetric division. Nature Communications 6: 8381.

Rabbit anti-Baz antibody was generated using the same peptide sequencing described in “Ohshiro T., et al., Nature 2000 Nov 30;408(6812):593-6”

Rabbit anti-Par6 antibody was generated using the same peptide (328-351aa of DmPar-6) described in “Petronczki M and Knoblich JA, Nat Cell Biol 2001 Jan;3(1):443-9”

Rabbit anti-Pon antibody was generate against the same peptide (13-43aa) described in “Lu B et al., Cell. 1998 Oct 16(95 (2):225-35”

Rabbit anti-aPKC C20 was described in “Wodarz A et al., J Cell Biol. 2000 Sep 18;150(6): 1361-74”

Chick anti-GFP was described in “Chai P, et al., PLoS Biol. 2013; 11(2): e1001494”

Anti-Numb antibody was a gift of Xiaohang Yang

Eukaryotic cell lines

Policy information about cell lines

Cell line source(s)	COS7 and HEK293T cells (from ATCC) were used in this study
Authentication	The cell lines were not authenticated.
Mycoplasma contamination	No mycoplasma contamination was detected in COS7 cells used in this study.
Commonly misidentified lines (See ICLAC register)	No commonly misidentified cell lines were used.
